# On some features characterizing the plasmasphere–magnetosphere–ionosphere system during the geomagnetic storm of 27 May 2017

**DOI:** 10.1186/s40623-019-1056-0

**Published:** 2019-07-17

**Authors:** Michael Pezzopane, Afredo Del Corpo, Mirko Piersanti, Claudio Cesaroni, Alessio Pignalberi, Simone Di Matteo, Luca Spogli, Massimo Vellante, Balazs Heilig

**Affiliations:** 1 Istituto Nazionale di Geofisica e Vulcanologia, Via di Vigna Murata 605, 00143 Rome, Italy; 2 Department of Physical and Chemical Sciences, University of L’Aquila, Via Vetoio, 67100 L’Aquila, Italy; 3 National Institute of Nuclear Physics, University of “Tor Vergata”, Via della ricerca scientifica 1, 00133 Rome, Italy; 4 SpacEarth Technology, Via di Vigna Murata 605, 00143 Rome, Italy; 5 Mining and Geological Survey of Hungary, Columbus Street 17-23, Budapest, 1145 Hungary

**Keywords:** Geomagnetic storm, Magnetopause motion, Magnetopause crossing, Plasmasphere dynamics, Geomagnetic field line resonances, Ionospheric currents, IRI UP method, Total electron content

## Abstract

This paper presents how the magnetosphere–plasmasphere–ionosphere system was affected as a whole during the geomagnetic storm peaking on 27 May 2017. The interplanetary conditions, the magnetospheric response in terms of the magnetopause motion, and the ionospheric current flow pattern were investigated using data, respectively, from the WIND spacecraft, from GOES15, GOES13, THEMIS E, THEMIS D and THEMIS A satellites and from the INTERMAGNET magnetometer array. The main objective of the work is to investigate the plasmaspheric dynamics under disturbed conditions and its possible relation to the ionospheric one; to reach this goal, the equatorial plasma mass densities derived from geomagnetic field line resonance observations at the European quasi-Meridional Magnetometer Array (EMMA) and total electron content values obtained through three GPS receivers close to EMMA were jointly considered. Despite the complexity of physical mechanisms behind them, we found a similarity between the ionospheric and plasmaspheric characteristic recovery times. Specifically, the ionospheric characteristic time turned out to be ~ 1.5 days, ~ 2 days and ~ 3.1 days, respectively, at *L* ~ 3, *L* ~ 4 and *L* ~ 5, while the plasmaspheric one, for similar *L* values, ranged from ~ 1 day to more than 4 days.
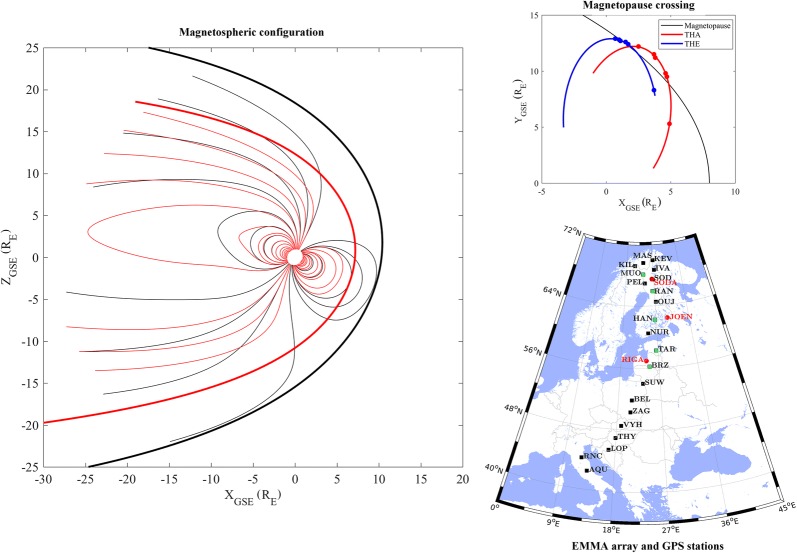

## Introduction

The coupling among the solar wind (SW), the magnetosphere and the ionosphere represents an important subject of scientific interest, in particular in the Space Weather context. In this process, the SW transfers energy to the magnetosphere by means of two principal mechanisms: the magnetic reconnection at the magnetopause in the dayside region [originally proposed by Dungey (1961)] and the viscous-like interaction generated by micro- or macro-instabilities [as suggested by Axford and Hines (1961)]. On the other hand, the magnetosphere and the ionosphere, strictly connected mainly through magnetic field-aligned processes, can exchange energy, momentum and particles (Kamide and Baumjohann 1993; Chappell 2015). Basically, three main processes (Blanc 1988) regulate the magnetosphere–ionosphere interaction: (1) the transmission of electric fields (Kikuchi 2014; Kikuchi and Hashimoto 2016), (2) the flows of electric charges by means of field-aligned currents (FACs; Lyons 2013; Baumjohann 1982) and (3) the precipitation and/or outflow of particles (Yau and André 1997; Schunk 2000; Longden et al. 2008; Newell et al. 2009). Additional and relevant features arise during geomagnetic storms (GSs) when the injection, transport and loss of charged particles related to the ring current play a major role in the dynamics of the circumterrestrial environment.

Concerning the ionosphere, its perturbations due to an increased dissipation of the solar wind energy represent still a challenging topic. These disturbances, called ionospheric storms, affect significantly the global morphology of the ionosphere and represent an important feature of the complex dynamics characterizing the solar–terrestrial relations. In addition, from the application point of view, they might highly degrade radio communications and satellite positioning (Park et al. 2016). However, in spite of the significant effort done so far to study ionospheric storms (Prölss 1995; Buonsanto 1999; Förster and Jakowski 2000; Mendillo 2006; Alfonsi et al. 2013; Borries et al. 2015; Cesaroni et al. 2017; Spogli et al. 2016; Greer et al. 2017; Habarulema et al. 2017; Heine et al. 2017), many features remain poorly understood as well as there are still many open questions, which testifies the complexity of the phenomenon.

Basically, during disturbed conditions, the electron density in the ionosphere can either increase or decrease, giving rise to positive and negative ionospheric storms, respectively. Positive storms can be caused by traveling atmospheric disturbances, large-scale changes in the wind circulation, magnetospheric convection and an expansion of the polar ionization enhancement; instead, negative storms can be caused by modifications in the composition of the neutral gas and equatorward displacement of the trough region (Prölss 1995; Mendillo 2006). Furthermore, longitudinal and latitudinal asymmetries often characterizing ionospheric storms, whose effects may vary considerably from one event to another, indicate that their global morphology is really complex. An additional aspect of interest is the switch from negative to positive storm effects in the upper F region, which makes a description of the disturbed topside ionosphere somewhat complicated (Reddy et al. 1967; Prölss 1995; Mendillo 2006; Tam et al. 2017).

An important aspect characterizing the dynamics of the inner magnetosphere is the plasma transport between the ionosphere and the plasmasphere. The particle exchange between the plasmasphere and the underlying ionosphere is continuous and controlled by the ambipolar diffusion along the field lines (Chappell 2015 and references therein). During daytime, the plasma density in the flux tubes gradually increases because of the particle diffusion from the ionosphere. After sunset, this process rapidly stops due to the decrease in the ionospheric charge content, which results in a downward flux of particles that causes plasmasphere depletion.

For very prolonged quiet geomagnetic conditions, the plasmasphere can reach a condition of saturation determined by diffusive equilibrium with the ionosphere. Anyhow, it is during disturbed geomagnetic conditions that the refilling process plays a key role in the dynamics of the inner magnetosphere. Several studies (e.g., Sandel et al. 2003; Spasojević et al. 2003, 2004; Abe et al. 2006; Kim et al. 2007) highlighted that the plasmasphere is highly dynamic and that the plasmapause can assume very complex configurations during periods of high geomagnetic activity, e.g., geomagnetic storms. In the aftermath of the disturbance, the outer plasmasphere is eroded due to an enhanced convection (e.g., Nishida 1966). During the recovery phase, the depleted flux tubes slowly recover to their initial condition thanks to an enhanced refilling from the ionosphere (e.g., Carpenter and Lemaire 1997). The contraction of the plasmasphere, caused by the enhanced convection, takes place rather quickly (on timescales of few hours) (e.g., Goldstein et al. 2003), while the refilling process is much longer (of the order of days) (e.g., Rasmussen et al. 1993; Reinisch et al. 2004; Dent et al. 2006; Sandel and Denton 2007; Piersanti et al. 2017b).

Another phenomenon occurring during GSs, different from the erosion driven by an enhancement of the convection, is the plasmasphere depletion (Park 1973; Chi et al. 2000; Clilverd et al. 2000; Wang et al. 2013). The plasmaspheric depletion (with density reductions of the order of a factor ~ 2–3) is often seen concurrently with ionospheric negative storms, as highlighted by Villante et al. (2006) and Wang et al. (2013); both works suggest that the plasmaspheric depletion is caused by a reduced upward flux from the perturbed ionosphere. On the other hand, model simulations by Clilverd et al. (2000) show that reasonable storm-time changes in thermospheric parameters, as well as possible **E **× **B** (where **E** is the ionospheric electric field and **B** is the geomagnetic field) plasma drifts to higher *L*-shells, cannot account for the observed plasmaspheric depletion. Therefore, this aspect definitely needs further investigations and is the main focus of the present paper, in the framework of the interplanetary coronal mass ejection (ICME) occurred on 27 May 2017.

Specifically, to analyze the plasmasphere depletion, the equatorial plasma mass densities derived from geomagnetic field line resonance (FLR) observations at the European quasi-Meridional Magnetometer Array (EMMA; Lichtenberger et al. 2013; Del Corpo et al. 2018), and vertical total electron content (*vTEC*) values obtained through three GPS receivers close to EMMA, are jointly considered with the specific aim to investigate whether the variation of ionospheric quantities is related somehow to the plasmasphere dynamics. In detail, the International Reference Ionosphere UPdate (IRI UP) procedure, which has been recently developed by Pignalberi et al. (2018a, b), is used to get, at the same GPS receiver locations, an estimation of the bottom *vTEC* (hereafter *bTEC*); in this way, an estimation of the top *vTEC* (hereafter *tTEC*) is obtained as the difference between *vTEC* and *bTEC*. The reason of this discrimination is twofold: On the one hand, to investigate the different response of these quantities to the ICME under investigation; on the other hand, to investigate possible connections between their variations and the plasmasphere dynamics.

Moreover, due to the good data coverage in terms of both satellites and ground-based observations, we gave a global picture of the plasmasphere–magnetosphere–ionosphere system response to the ICME: We analyzed the interplanetary conditions through data from the WIND spacecraft; we evaluated the magnetosphere response using measurements from GOES15, GOES13, THEMIS E, THEMIS D and THEMIS A satellites; we figured out the ionospheric current flow pattern using data from the INTERMAGNET ground magnetometer array.

The paper is organized as follows: “Data and methods” describes data and methods; “Interplanetary conditions” gives an interpretation of the interplanetary conditions; “Magnetospheric response” focuses on the magnetospheric and plasmaspheric response; “Ionospheric response” focuses on the ionospheric response; discussion and conclusions are finally the subject of “Discussion and conclusions”.

## Data and methods

The interplanetary medium conditions between 25 May and 2 June 2017 have been analyzed using SW parameters as measured by WIND–SWE (Ogilvie et al. 1995) and interplanetary magnetic field (IMF) measurements as recorded by WIND–MFI (Lepping et al. 1995).

The magnetosphere response, in terms of both the magnetopause motion and the response at geostationary orbit, is investigated by using magnetic field measurements recorded by different spacecrafts: GOES15, GOES13, THEMIS E, THEMIS D and THEMIS A.

**Fig. 1:**
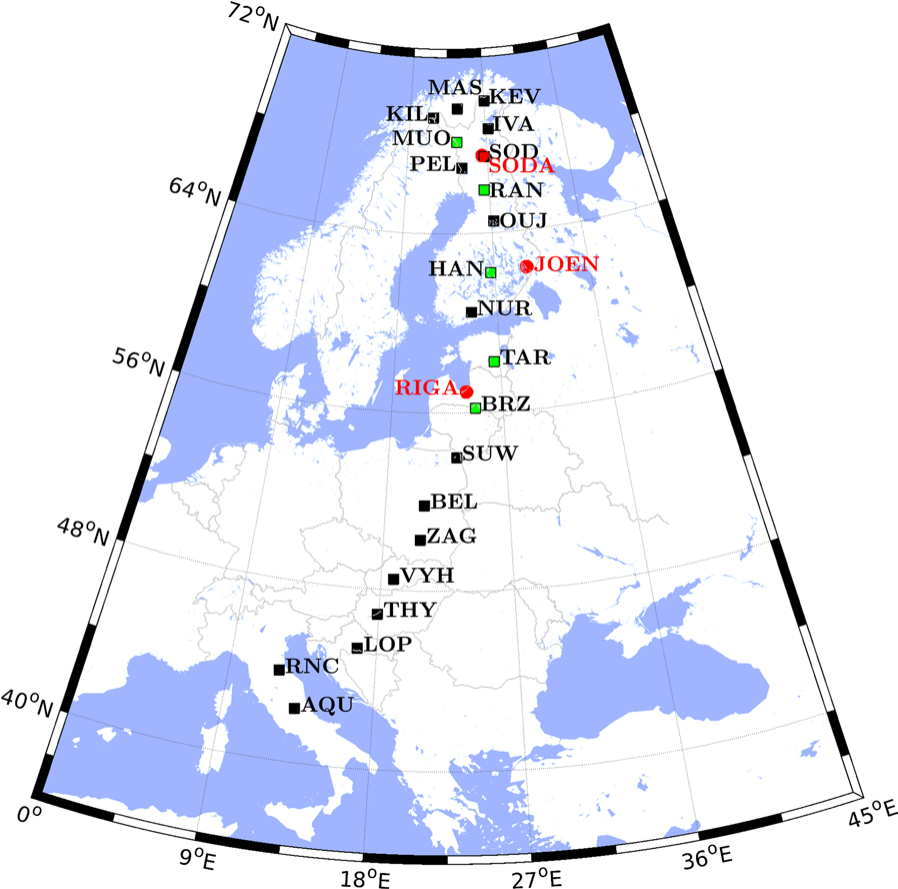
Map of the EMMA stations (square markers) used for the plasmasphere analysis. Green squares identify the station pairs used to describe in detail the plasmasphere dynamics at three different latitudes. Red circles are the positions of the global navigation satellite system receivers nearest to the considered station pairs and used for the ionospheric analysis described in “Ionospheric response in terms of vertical, top, and bottom TEC.” Coordinates are geographic. See Table 1 for additional info about the EMMA stations used in the analysis

The properties and the dynamics of the plasmasphere have been investigated by studying the spatial–temporal variation of the equatorial plasma mass density as deduced from ground-based measurements of geomagnetic FLR frequencies (Menk et al. 2014). The method compares the amplitude and phase spectra of ultra-low-frequency (ULF) measurements at pairs of magnetometer stations slightly separated in latitude to identify the resonance frequency of the field line midway between the two stations (Baransky et al. 1985; Waters et al. 1991). Once the FLR frequency is determined, the plasma mass density at the equatorial point of the field line can be inferred by solving the governing magnetohydrodynamic wave equation (Singer et al. 1981; Vellante et al. 2014a) and using proper models of both the magnetic field (Berube et al. 2006; Vellante et al. 2014a, b) and the plasma density distribution along the field line (Menk et al. 1999; Takahashi et al. 2004; Vellante and Förster 2006). Having available an array of ULF station pairs at different latitudes, the radial variation of the equatorial density can be derived. In this study, we applied the semi-automated procedure proposed by Del Corpo et al. (2018) to the 1-s data from the EMMA array, which allows to monitor the plasma mass density in the *L*-range 1.5–6.5. In this procedure, the magnetospheric field is described by the TS05 model (Tsyganenko and Sitnov 2005), and the plasma mass density *ρ* along the field line is assumed to vary as *ρ* = *ρ*_0_ (*r*_0_/*r*) (Vellante and Förster 2006), where *r* is the geocentric distance and the subscript “0” identifies values at the magnetic equator. Resonance frequencies are determined through the fast Fourier transform using a sliding time window of 2 h advanced at half an hour time step. The event presented in this study is part of the data set analyzed by Del Corpo et al. (2018), and more details about the method can be found in that paper. The location of the considered magnetic stations is shown in Fig. 1, and Table 1 gives additional information about the corrected geomagnetic coordinates and the associated *L* value evaluated at an altitude of 120 km for 1 January 2017.

**Table 1:** List of EMMA geomagnetic stations used for the plasmaspheric analysis

Station	Code	CGM longitude (°)	CGM latitude (°)	*L*
Kevo	KEV	108.3	66.7	6.5
Masi	MAS	105.4	66.5	6.4
Kilpisjärvi	KIL	102.3	66.2	6.3
Ivalo	IVA	107.7	65.5	5.9
Muonio	MUO	104.3	65.0	5.7
Sodankylä	SOD	106.5	64.3	5.4
Pello	PEL	104.1	63.9	5.3
Raniu	RAN	105.5	62.8	4.9
Olujärvi	OUJ	105.6	61.3	4.4
Hankasalmi	HAN	104.1	59.0	3.8
Nurmijärvi	NUR	101.8	57.2	3.5
Tartu	TAR	102.6	54.8	3.1
Birzai	BRZ	100.5	52.6	2.8
Suwalki	SUW	98.6	50.1	2.5
Belsk	BEL	95.6	47.7	2.3
Zagorzyce	ZAG	95.4	45.9	2.1
Vyhne	VYH	93.5	43.8	2.0
Tihany	THY	92.4	41.9	1.8
Lonjsko Polje	LOP	91.1	40.0	1.7
Ranchio	RNC	86.7	38.1	1.7
L’Aquila	AQU	87.5	36.2	1.6

The corrected geomagnetic (CGM) coordinates and *L* values are evaluated at an altitude of 120 km for 1 January 2017

**Fig. 2:**
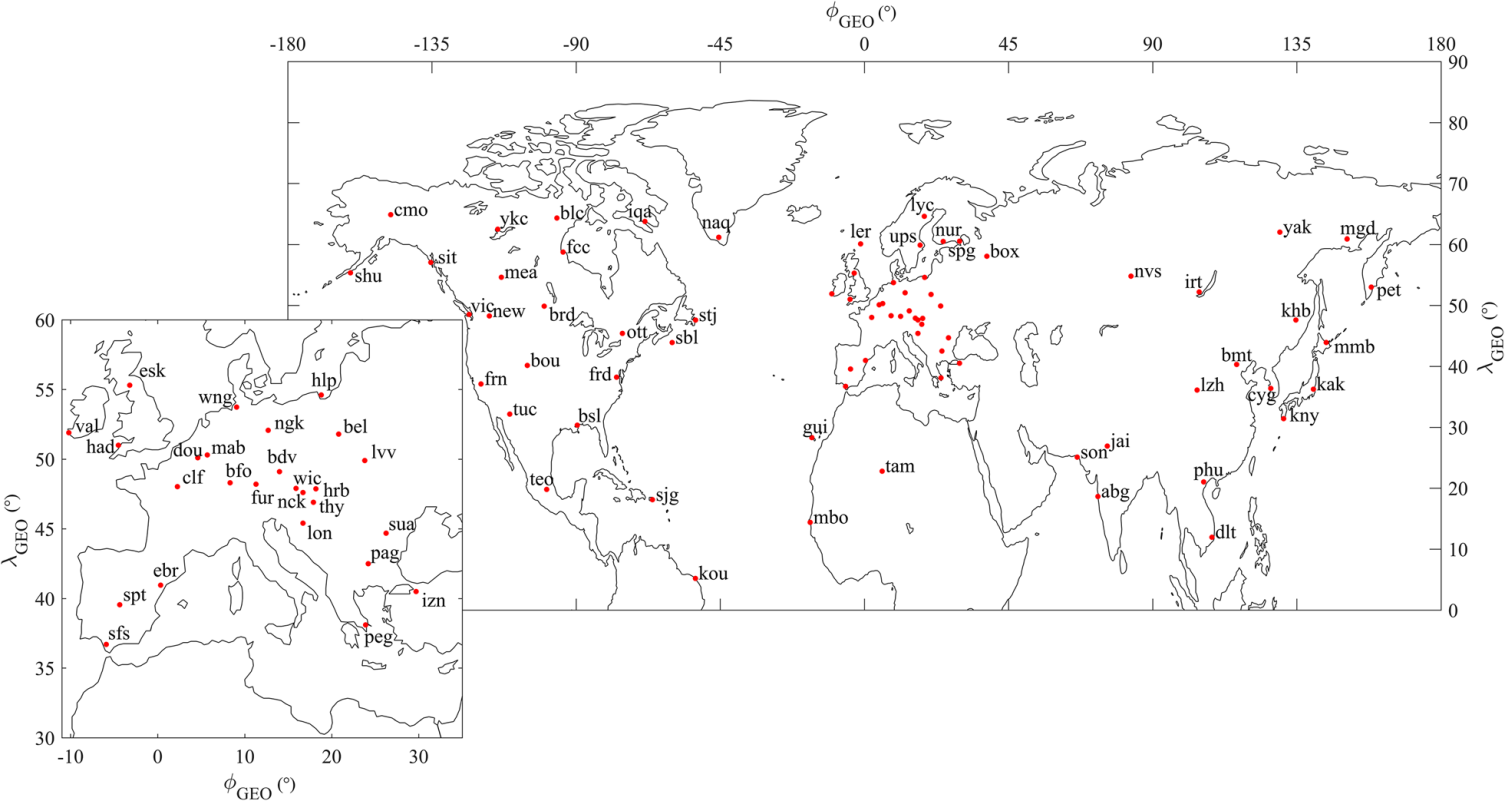
Name and location (red dots), in terms of geographical coordinates, of the 75 INTERMAGNET stations used for the evaluation of the ionospheric current pattern associated with the SI

A GS is typically initiated by a sudden impulse (SI), caused by an interplanetary shock (IS) impinging on the magnetopause and compressing the magnetosphere. The ionospheric response to the ICME impact into the magnetosphere is studied by reconstructing the ionospheric current flow pattern produced during the SI, the so-called DP 2-type current systems. They consist in a double-cell structure, induced by the SW pressure enhancement (Araki 1994; Piersanti and Villante 2016; Carter et al. 2016). The current understanding proposes that the total disturbance field (*D*_SI_), observed during an SI, can be divided into two different contributions: *D*_SI_ = DL + DP (Araki 1994), where DL is a contribute of magnetospheric origin dominant at low latitudes (L stands right for low latitudes), while DP is a contribute of ionospheric origin prevailing at high latitudes (P stands right for polar latitudes). At low latitudes, the DP field shows positive variations along the north–south magnetic component (*H*) and almost negligible variations along the east–west magnetic component (*D*). In addition, the amplitudes along *H* are greater around local noon. Araki (1994) showed that the DP field is constituted by a double-pulse structure: a preliminary impulse (PI) and a main impulse (MI), both generated by the coupling between FACs and ionospheric currents (Araki et al. 2009). Recently, a new model to infer the DP 2-cell ionospheric current for both PI and MI has been developed by Piersanti and Villante (2016), who derived the DL field by comparing the magnetospheric field observations with the TS05 model previsions. They showed that the DP field is the residual part between ground observations and the estimated DL field. To evaluate the PI_IC_ and MI_IC_ current flow pattern of ionospheric origin (the subscript IC stands right for ionospheric contribution) associated with the SI occurred on 27 May 2017, we applied the Piersanti and Villante (2016) method to 75 ground magnetic INTERMAGNET observatories in the northern hemisphere (Fig. 2). We evaluated the DL field as the *B*_CF+R_ field output by the TS05 model (the subscript CF standing for the Chapman–Ferraro current and the subscript R standing for the ring current) and then the DP field as the residual between ground magnetic observations and the estimated DL field along both *H* and *D* components.

The ionospheric response is also investigated by analyzing the *vTEC*, *tTEC* and *bTEC* during the entire magnetic disturbed period. Receiver-independent exchange (RINEX) files from the EUREF Permanent GNSS Network (http://www.epncb.oma.be/) containing GPS code and carrier-phase observables acquired every 15 min (from 00:00 to 23:45 UT of each day), from 26 May to 2 June 2017, were used to obtain calibrated *vTEC* values at SODA (67.4°N, 26.4°E), JOEN (62.4°N, 30.1°E) and RIGA (56.9°N, 24.1°E), by applying the Ciraolo et al. (2007) and Cesaroni et al. (2015) method. We focused right on these receivers because they are the closest ones to the pairs of magnetometers MUO–RAN, RAN–HAN and TAR–BRZ of the EMMA array, respectively, which are used to describe the latitudinal dependence of the plasmasphere dynamics. The single-station method proposed by Ciraolo et al. (2007) and Cesaroni et al. (2015) tries to estimate both the phase ambiguity by applying the so-called leveling procedure and, for each arc of observation (i.e., for each satellite–receiver pair), biases and all nonzero mean errors (e.g., multipath) that can affect slant *TEC* (*sTEC*) values along the satellite–receiver line of sight:

1 $$\tilde{L}_{\text{arc}} = sTEC + \beta_{\text{arc}} ,$$

where $\tilde{L}_{\text{arc}}$ represents the “levelled” carrier-phase observable along each arc of observation and $\beta_{\text{arc}}$ is the error associated with the same arc including all biases introduced by both the satellite and the receiver. In order to estimate *sTEC*, a least-square method is applied by defining:

2 $$sTEC = vTEC\left( {modip,{\text{LT}}} \right)\cos \chi$$

where *modip* is the modified dip latitude (Rawer et al. 1981), LT is the local time and cos(*χ*) represents the mapping function described in Mannucci et al. (1998). This allows to calculate the value of *vTEC* associated with every couple (*modip*, LT), including the one representing the Ionospheric Pierce Point at the vertical of the receiver. Values of *bTEC* at the same locations were calculated through the IRI UP method proposed by Pignalberi et al. (2018a, b). The purpose of IRI UP is to update the IRI model (Bilitza et al. 2014) through the assimilation of the ionospheric characteristics *fo*F2 (the F2-layer critical frequency) and *M*(3000)F2 (the propagation factor), registered by a European ionosonde network. First, such measurements are used to evaluate, at each ionosonde location, the updated (effective) values of both the ionospheric index *IG*_12_ (Liu et al. 1983) and the sunspot number *R*_12_, identified as *IG*_12eff_ and *R*_12eff_ (Houminer and Soicher 1996). Secondly, this discrete dataset of effective indices is used to generate two-dimensional European maps of these indices by applying the Universal Kriging method (Kitanidis 1997). Computed maps of effective indices are then used as input for the IRI model to obtain over the European region a three-dimensional updated representation of the electron density (Pietrella et al. 2018; Pignalberi et al. 2018c). *bTEC* values are then calculated over the three considered locations, SODA, JOEN and RIGA, by numerically integrating the following equation:

3 $$bTEC = \int\limits_{{h_{\text{b}} }}^{{hm{\text{F}}2}} {N_{\text{e}} (h)_{\text{bottom}} {\text{d}}h} ,$$

where *h*_b_ is the height of the base of the ionosphere and *N*_e_(*h*)_bottom_ is the bottom side electron density profile calculated by the IRI UP method. To accomplish this task, ionospheric characteristics recorded by the following European ionospheric stations were considered: Athens (Greece, 38.0°N, 23.5°E), Chilton (UK, 51.5°N, 0.6°W), Dourbes (Belgium, 50.1°N, 4.6°E), Fairford (UK, 51.7°N, 1.5°W), Juliusruh (Germany, 54.6°N, 13.4°E), Moscow (Russia, 55.5°N, 37.3°E), Nicosia (Cyprus, 35.0°N, 33.2°E), Pruhonice (Czech Republic, 50.0°N, 14.6°E), Rome (Italy, 41.8°N, 12.5°E), Roquetes (Spain, 40.8°N, 0.5°E), San Vito (Italy, 40.6°N, 17.8°E), Tromso (Norway, 69.6°N, 19.2°E) and Warsaw (Poland, 52.2°N, 21.1°E). Since the sounding repetition rate of most of ionosondes was set to 15 min, ionograms recorded every 15 min (at minutes 00, 15, 30 and 45 of each hour) have been used. This is why only *vTEC* values every 15 min, from 00:00 to 23:45 UT, were considered as well. Ionosonde data come from the Digital Ionogram DataBASE (Reinisch and Galkin 2011) through the SAO Explorer software (https://ulcar.uml.edu/SAO-X/SAO-X.html). All the considered ionospheric stations are equipped with a DPS4 digisonde (Bibl and Reinisch 1978), except Rome, for which data recorded by an AIS-INGV ionosonde (Zuccheretti et al. 2003) were considered, and Warsaw, equipped with a VISRC2 ionosonde (Pezzopane et al. 2010). Even though ionograms recorded by digisondes are autoscaled by the Automatic Real-Time Ionogram Scaler with True height system (Reinisch and Huang 1983; Reinisch et al. 2005), and ionograms recorded at Rome and Warsaw are autoscaled by the Autoscala system (Pezzopane and Scotto 2005, 2007; Scotto et al. 2012), the whole ionogram dataset of each station was visually inspected and validated. Once *vTEC* and *bTEC* had been calculated, *tTEC* over SODA, JOEN and RIGA was obtained as the difference between *vTEC* and *bTEC*.

## Interplanetary conditions

**Fig. 3:**
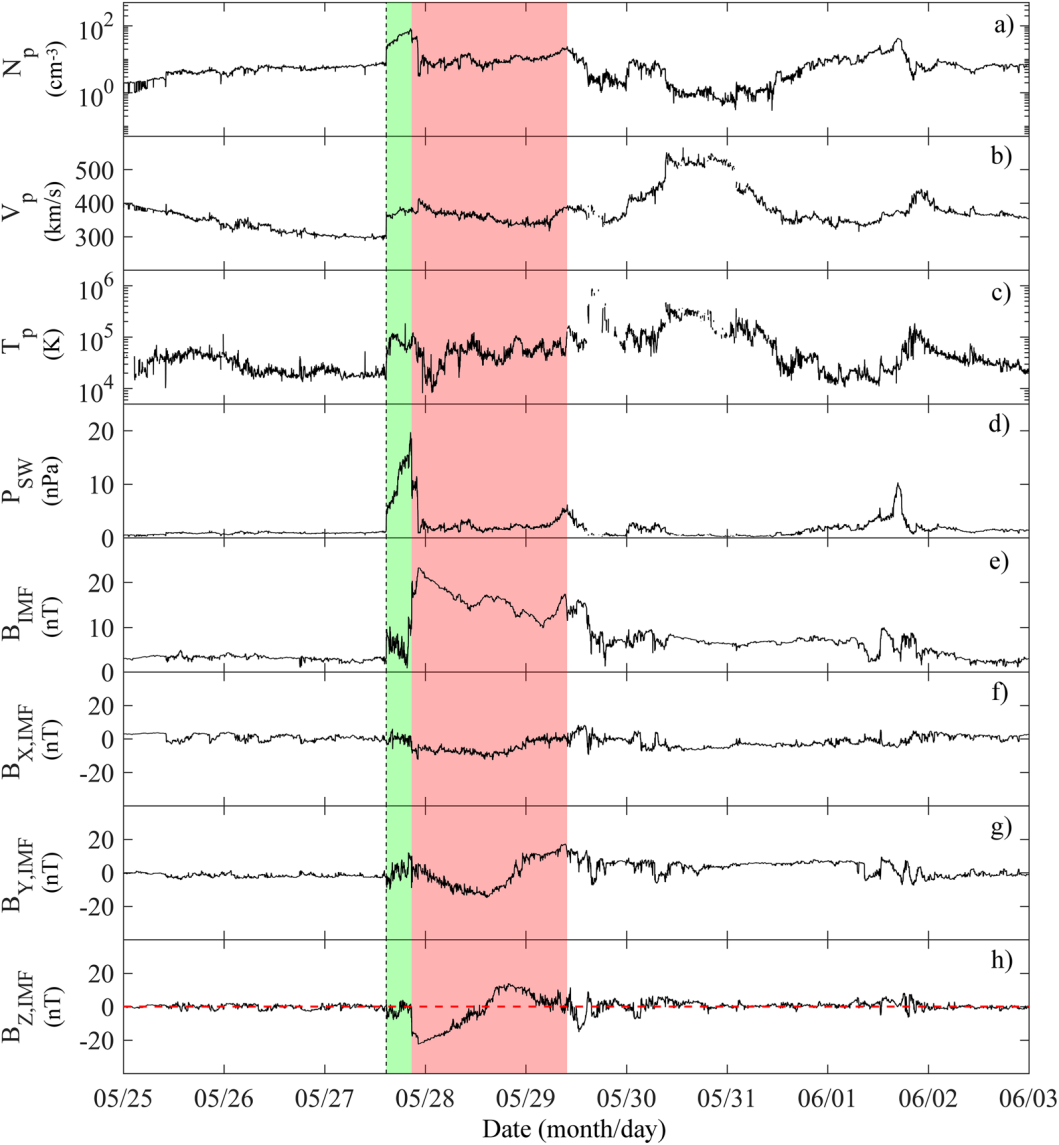
Solar wind parameters between 25 May and 2 June 2017 observed by WIND (at first Lagrangian point), in UT: **a** proton density, **b** proton velocity, **c** proton temperature, **d** pressure, **e** IMF intensity, **f**–**h** IMF *x*, *y*, *z* components in the GSE coordinate system. The vertical black dashed line indicates the arrival of the interplanetary shock, on 27 May 2017 at 14:41 UT. The green area beyond is the ICME sheath, while the red area corresponds to the magnetic cloud (ejecta interval)

Figure 3 shows that an IS (black dashed line), identified by a clear jump in the SW proton density (∆*N*_p_ ~ 14 cm^−3^, panel a), proton velocity (∆*V*_p_ ~ 70 km/s, panel b), proton temperature (∆*T*_p_ ~ 4 × 10^4^ K, panel c), pressure (∆*P*_SW_ ~ 3.5 nPa, panel d) and IMF (∆*B*_IMF_ ~ 5 nT, panel e), associated also with a southward IMF component (*B*_*z*,__IMF_ ~ −4 nT, panel h), passed the WIND spacecraft (*X*_GSE_ ~ 204 R_E_, *Y*_GSE_ ~ − 53.0 R_E_, and *Z*_GSE_ ~ −5 R_E_, where GSE stands for the geocentric solar ecliptic system and R_E_ is the Earth radius) at 14:41 Universal Time (UT) on 27 May 2017. A sheath region (highlighted in green in Fig. 3) follows until ~ 20:46 UT, when *N*_p_ reaches the highest value of ~ 84 cm^−3^, and when the passage of an ICME is recognized. Using the Rankine–Hugoniot relations (Vias and Scudder 1986; Szabo 1994), we estimated the shock normal orientation, with *Φ*_GSE_ ~ 162° (the angle measured from the *X*_GSE_ direction to the projection of the shock normal to the *XY*_GSE_ plane) and *Θ*_GSE_ ~ 25.5° (the elevation with respect to the *XY*_GSE_ plane), and a value of the shock speed *V*_sh_ of about 349 km/s. Then, the decrease in the proton density and temperature (shown, respectively, in panels a and c of Fig. 3), in conjunction with the increase in the strength of the IMF (panel e), coupled with its smooth rotation (panels f, g, and h), is the signature of a magnetic cloud (Zurbuchen and Richardson 2006). The boundaries of this structure (highlighted in red in Fig. 3) have been determined by the sharp variations in the IMF strength at ~ 20:46 UT on 27 May 2017 and at ~ 9:50 UT on 29 May 2017, respectively.

## Magnetospheric response

### Magnetospheric sudden impulse

**Fig. 4:**
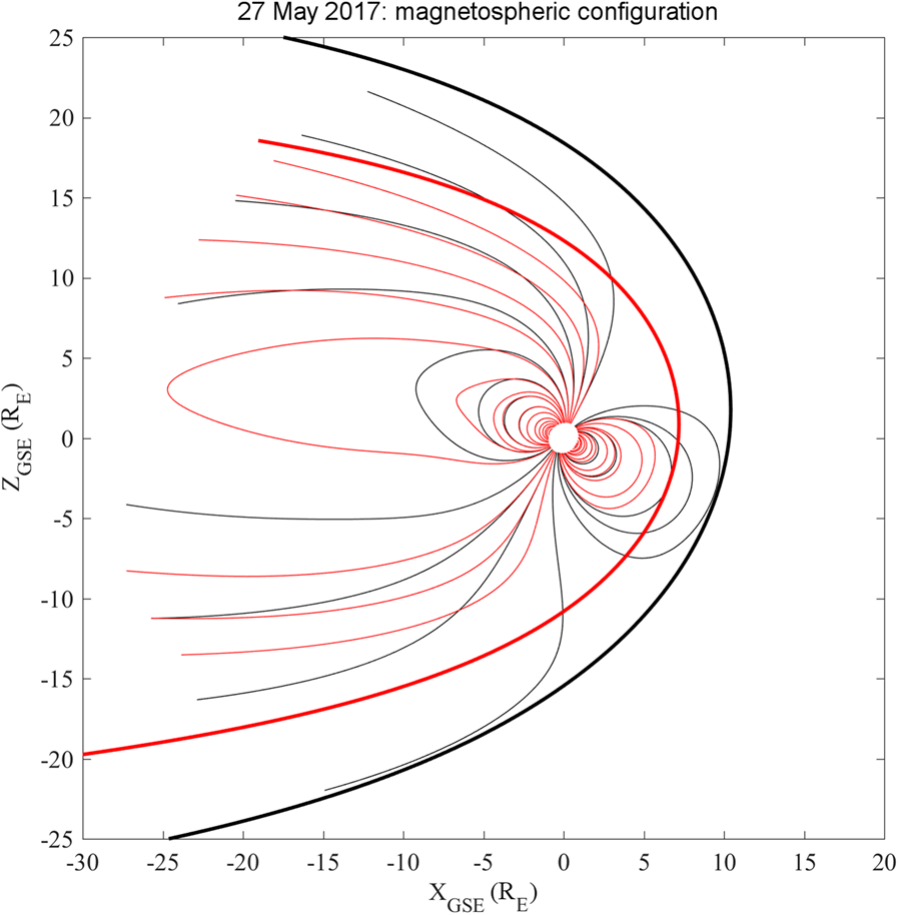
Magnetospheric field lines configuration as evaluated by the TS05 model before (black lines) and after (red lines) the IS impact. Black and red thick curves represent the magnetopause configuration before and after the IS passage, respectively

Figure 4 shows the magnetopause profile and the magnetospheric field lines configuration during the IS passage as expected by the TS05 model, which predicts an inward motion of the magnetopause nose from ~ 10.2 R_E_ (black dotted curve) to ~ 7.2 R_E_ (red dotted curve). The solar wind parameters input to the TS05 model are reported in Table 2.

**Table 2:** Solar wind parameters input to the TS05 model to obtain Fig. 4

	SW pressure (nPa)	Sym-H (nT)	*B*_*y*,IMF_ (nT)	*B*_*z*,IMF_ (nT)
Before the IS	0.5	8.1	− 0.02	− 1.8
After the IS	8.5	20.5	1.40	− 5.2

**Fig. 5:**
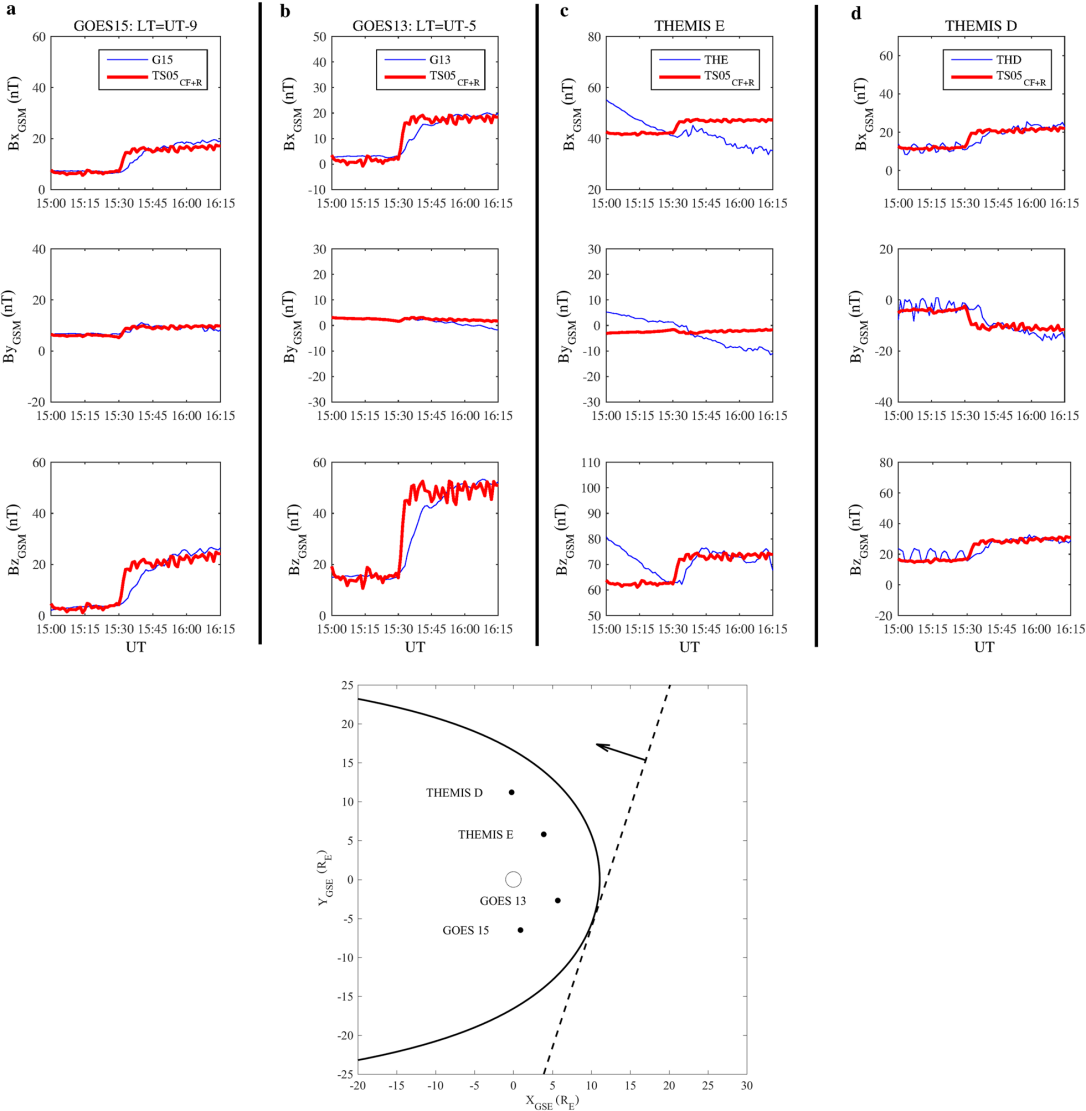
Comparison between *B*_*x*GSM_ (upper panels; GSM stands for the geocentric solar magnetic system), *B*_*y*GSM_ (middle panels) and *B*_*z*GSM_ (lower panels) observations (blue lines) and the *B*_CF+R_ field output by the TS05 model (red lines; the subscript CF + R means that the TS05 model has been run by considering only the magnetopause and ring current contributions), for GOES15 (column **a**), GOES13 (column **b**), THEMIS E (column **c**) and THEMIS D (column **d**) on 27 May 2017 between 15:00 and 16:15 UT. The bottom panel shows the position of GOES13, GOES15, THEMIS D and THEMIS E (black full circles), the model-calculated magnetopause location before the IS passage (black thick line) and the direction of the IS front (black dashed lines) impinging the magnetopause. Black empty circle represents the Earth’s position

Figure 5 shows the magnetospheric response, as recorded by GOES15, GOES13, THEMIS E and THEMIS D, compared with that predicted by the TS05 model, considering only the magnetopause and ring current contributions. Panels from (a) to (d) display GOES15, GOES13, THEMIS E and THEMIS D measurements, respectively. To give the reader an overall picture of the situation, the bottom panel of Fig. 5 shows also the position of each satellite, the model-calculated magnetopause before the IS passage and the direction of the IS front. Both GOES15 and GOES13, located, respectively, at *X*_GSE_ ~ 0.9 R_E_, *Y*_GSE_ ~ −6.5 R_E_, *Z*_GSE_ ~ 0.8 R_E_ and *X*_GSE_ ~ 5.7 R_E_, *Y*_GSE_ ~ −2.7 R_E_, *Z*_GSE_ ~ −1.8 R_E_, corresponding to 6:30 LT and 10:30 LT, observed an SI characterized by a slow rise in the magnetic field components.

In the post-noon magnetosphere, THEMIS E, located at *X*_GSE_ ~ 4 R_E_, *Y*_GSE_ ~ 6 R_E_ and *Z*_GSE_ ~ −3 R_E_, observed the SI almost at the same GOES time, while THEMIS D, located at *X*_GSE_ ~ −0.23 R_E_, *Y*_GSE_ ~ 11 R_E_ and *Z*_GSE_ ~ −2 R_E_, after a persistent wave activity in each magnetic field component interrupted by the shock impact, shows a clear jump at 15:36 UT three minutes later than other satellites. This can be related to the disturbance that, according to the estimated impact point, propagates from the pre-noon region to the post-noon region.

Indeed, the jump in the *B*_*z*_ component of the magnetospheric field is greater (∆*B*_*z*GSM_ ~ 29 nT, where GSM stands for the geocentric solar magnetic system) at GOES13 (the nearest to the shock impact point in the pre-noon region) than at GOES15 (dawn region, ∆*B*_*z*GSM_ ~ 16 nT) and THEMIS E (post-noon region, ∆*B*_*z*GSM_ ~ 12 nT) and assumes the lowest value at THEMIS D (dusk region, ∆*B*_*z*GSM_ ~ 8 nT). These observations are in agreement with the results shown by Villante and Piersanti (2008) when analyzing disturbed periods between 2000 and 2004.

### Magnetopause crossings

**Fig. 6:**
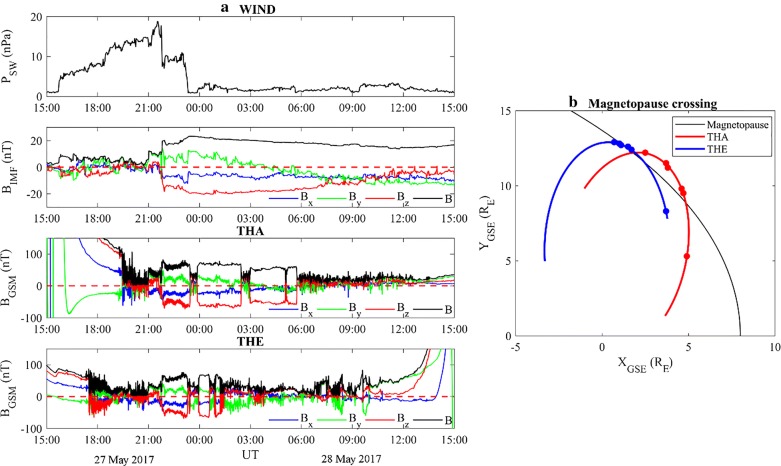
**a** From the top: the dynamic pressure of the solar wind as measured by WIND, the strength and the three components of IMF at WIND and of the magnetospheric field at THEMIS A (THA) and THEMIS E (THE), from 15:00 UT on 27 May 2017 to 15:00 UT on 28 May 2017. **b** The black curve shows the Shue et al. (1998) magnetopause profile for *P*_SW_ ~ 2 nPa and *B*_*z*,__IMF_ ~ −18 nT. Red and blue curves show the trajectories of THA and THE respectively, from ~ 17:00 UT on 27 May 2017 to ~ 13:00 UT on 28 May 2017 (full circles identify the magnetopause crossings)

Figure 6a shows, from the top, the dynamic pressure, the strength and the three components of the IMF as recorded by WIND (these data have been temporally moved forward of ~ 62 min), in comparison with the magnetospheric field as recorded by THEMIS A and THEMIS E 24 h beyond the SI. (THEMIS D data were not available for this time window.)

After the IS impact, during the period from ~ 17:29 UT on 27 May 2017 to ~ 05:44 UT on 28 May 2017, the magnetic field measured by THEMIS A and THEMIS E manifests sharp southward rotations of the *B*_*z*_ component (whose amplitude is of the order of the magnetic field strength) and strong variations of the other components. These features, as highlighted by Suvorova et al. (2005) and Dmitriev et al. (2005), are signature of multiple magnetopause crossings. This is strictly confirmed by a direct correspondence between the behavior of the magnetic field components and the behavior of the IMF. Table 3 reports the time and the position of both THEMIS A and THEMIS E for each identified magnetopause crossing.

**Table 3:** Magnetopause crossings for THEMIS A and THEMIS E: occurrence time (from 27 May 2017 at 17:29 UT to 28 May 2017 at 05:44 UT), GSE coordinates and distance from the Earth

Satellite	Date and Universal Time	(*X*_GSE_, *Y*_GSE_, *Z*_GSE_) (R_E_)	*r* (R_E_)
THEMIS A	27 May 2017 19:26	(4.9, − 5.3, − 3.9)	8.2
	27 May 2017 23:27	(4.7, 9.5, − 4.9)	11.7
	27 May 2017 23:51	(4.6, 9.8, − 4.9)	11.9
	28 May 2017 02:28	(3.8, 11.2, − 5.0)	12.8
	28 May 2017 02:56	(3.7, 11.5, − 4.9)	13.0
	28 May 2017 05:44	(2.5, 12.2, − 4.6)	13.3
THEMIS E	27 May 2017 17:29	(3.7, 8.3, − 3.5)	9.7
	27 May 2017 23:18	(1.7, 12.4, − 3.5)	13.0
	27 May 2017 23:50	(1.5, 12.6, − 3.4)	13.1
	28 May 2017 00:36	(1.1, 12.7, − 3.3)	13.2
	28 May 2017 00:52	(1.0, 12.8, − 3.1)	13.2
	28 May 2017 01:28	(0.7, 12.9, − 3.1)	13.3

Figure 6a highlights also that, after the IS signature, during the continuous increase in the dynamic pressure of the SW, THEMIS E and THEMIS A show large fluctuations in the magnetic field that identify their get in through the magnetosheath (at ~ 17:29 UT and at ~ 19:26 UT, respectively). On the other hand, at ~ 21:00 UT and at ~ 21:47 UT, when a sudden southward rotation of *B*_*z*,__IMF_ is observed by WIND, both THEMIS A and THEMIS E measure simultaneously a jump of the magnetic field strength. This suggests that both satellites were in the transition region, despite a huge drop characterizing the *P*_SW_. In fact, the erosion process due to the southward IMF rotation balances the negative *P*_SW_ variation and the consequent outward motion of the magnetopause.

At ~ 23:16 UT, a second fall of the *P*_SW_, coupled with a constant negative *B*_*z*,__IMF_, determines an outward motion of the magnetopause. In fact, both THEMIS E and THEMIS A observe new magnetopause crossings at ~ 23:18 UT and ~ 23:27 UT, respectively. In the following hours, the interplanetary parameters remain almost stable with *P*_SW_ ~ 2 nPa and *B*_*z*,__IMF_ ~ −18 nT, and the next crossings experimented by both THEMIS A and THEMIS E are the consequence of their outward orbital motion through the new stable magnetopause.

After the last magnetopause crossing, both THEMIS A (beyond ~ 05:44 UT on 28 May 2017) and THEMIS E (beyond ~ 01:28 UT on 28 May 2017) observations become highly dynamic. This may be related to a combined effect of their vicinity to the magnetosphere boundary and their orbital motion.

To summarize, Fig. 6b shows the magnetopause profile (black curve), as obtained by the Shue et al. (1998) model, the trajectories of THEMIS A (red curve) and THEMIS E (blue curve), and the corresponding magnetopause satellites crossings (identified by full circles). It is worth highlighting the good agreement between the magnetopause identified in the THEMIS E data and the one modeled by the Shue et al. (1998) model.

### Plasmasphere analysis

**Fig. 7:**
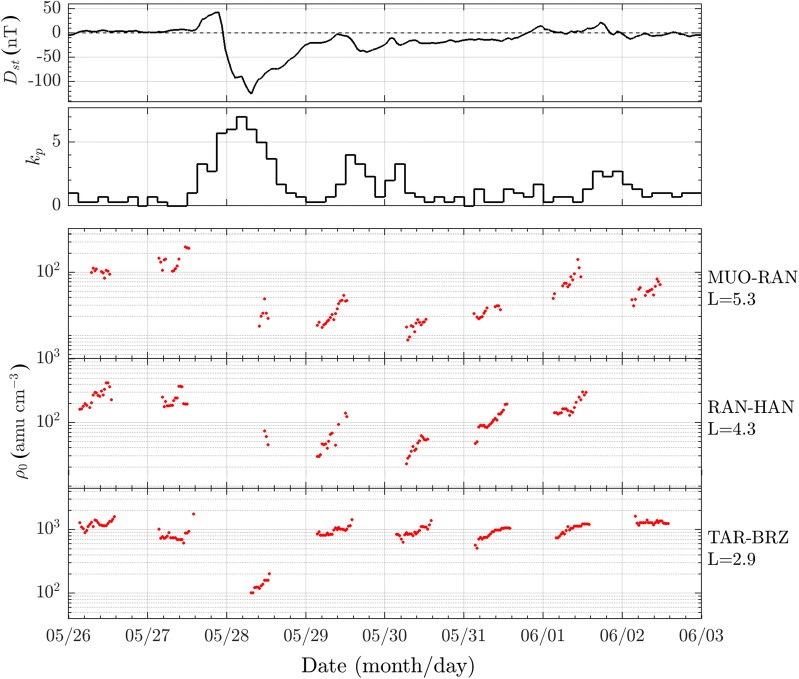
From the top: the *D*_st_ index, the *k*_p_ index and the equatorial plasma mass density at *L* = 5.3, 4.3 and 2.9, respectively, estimated from ULF frequencies obtained from the three EMMA station pairs MUO–RAN, RAN–HAN and TAR–BRZ. Values are in UT

Figure 7 shows the plasma mass density variations between 26 May and 2 June 2017 as inferred from three different station pairs (green squares in Fig. 1) at *L* = 2.9, 4.3, 5.3. We limited our analysis to hours when the field line footprints at 120 km altitude in both hemispheres were sunlit (approximately 03–13 UT). This condition ensures that the detected FLR frequencies correspond to half-wave standing field line oscillations, leading to a correct estimation of the mass density (Obana et al. 2015; Del Corpo et al. 2018). The same figure shows also the behavior of the geomagnetic *D*_st_ and *k*_p_ indices.

The storm under investigation was preceded by a period of very quiet geomagnetic activity conditions resulting in *k*_p_ ≤ 1 and |*D*_st_| < 10 nT for at least 3 days before the SI, which is clearly visible on 27 May 2017 at around 15:30 UT as a simultaneous increase of both indices. This prolonged quiet condition, only partially visible in Fig. 7, allows the plasmasphere to reach a state of quasi-saturation. As a result, the average densities at different *L* on 26 and 27 May 2017 show approximately the same values, with the notable exception at *L* = 2.9 where a density drop of about 30% is observed on 27 May 2017. This feature cannot be the result of an enhanced convection electric field and needs a further investigation to be explained which is out the scope of this work. In what follows, we use the 26 May 2017 to refer to saturated plasmasphere conditions for the period under study.

The main phase of the storm starts at the end of 27 May 2017 and the effects of the plasmasphere erosion are clearly visible on 28 May 2017. The density falls by a factor ranging between 5 and 10, although only few points are available for the station pairs at the highest latitudes.

During the recovery phase, the effects of both the refilling from the ionosphere and the convection electric field enhancement are visible. The former process produces the typical diurnal pattern in which the density monotonically increases during the day. Such trend is clearly visible on 29 May 2017 and is relatively more pronounced as the latitude increases. The latter process generally causes a decrease in the average level of the plasma density. An example is the density drop on 30 May 2017 at *L* = 4.3 and *L* = 5.3 following the enhanced convection as indicated by an increase in the *k*_p_ index between 29 and 30 May 2017. A similar effect is also visible on 2 June 2017 at *L* = 5.3. In general, the recovery of the plasmasphere to pre-storm conditions depends on both the latitude and the local time (e.g., Dent et al. 2006; Piersanti et al. 2017b). At *L* = 2.9, the recovery time is of the order of ~ 1 day, while at *L* = 4.3 is ~ 4 days and possibly even more at *L* = 5.3.

**Fig. 8:**
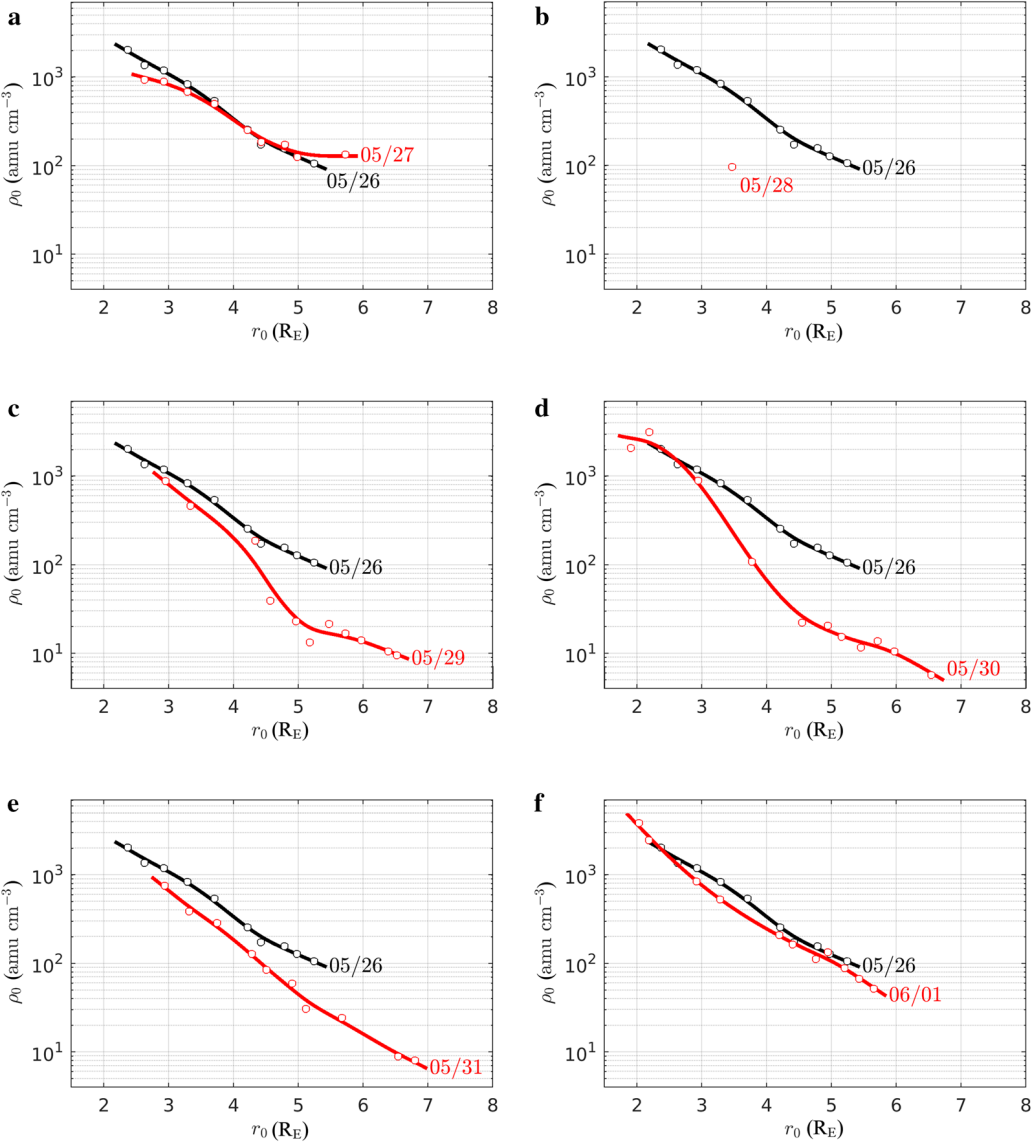
Radial profile of the equatorial plasma mass density (red curve in **a**–**f**) observed at 06:30 UT (~ 08:30 LT) between 27 May and 1 June 2017. For comparison, each panel shows also the density profile of 26 May 2017 (black curve). Solid lines are smoothed spline fits of experimental points (open circles)

**Fig. 9:**
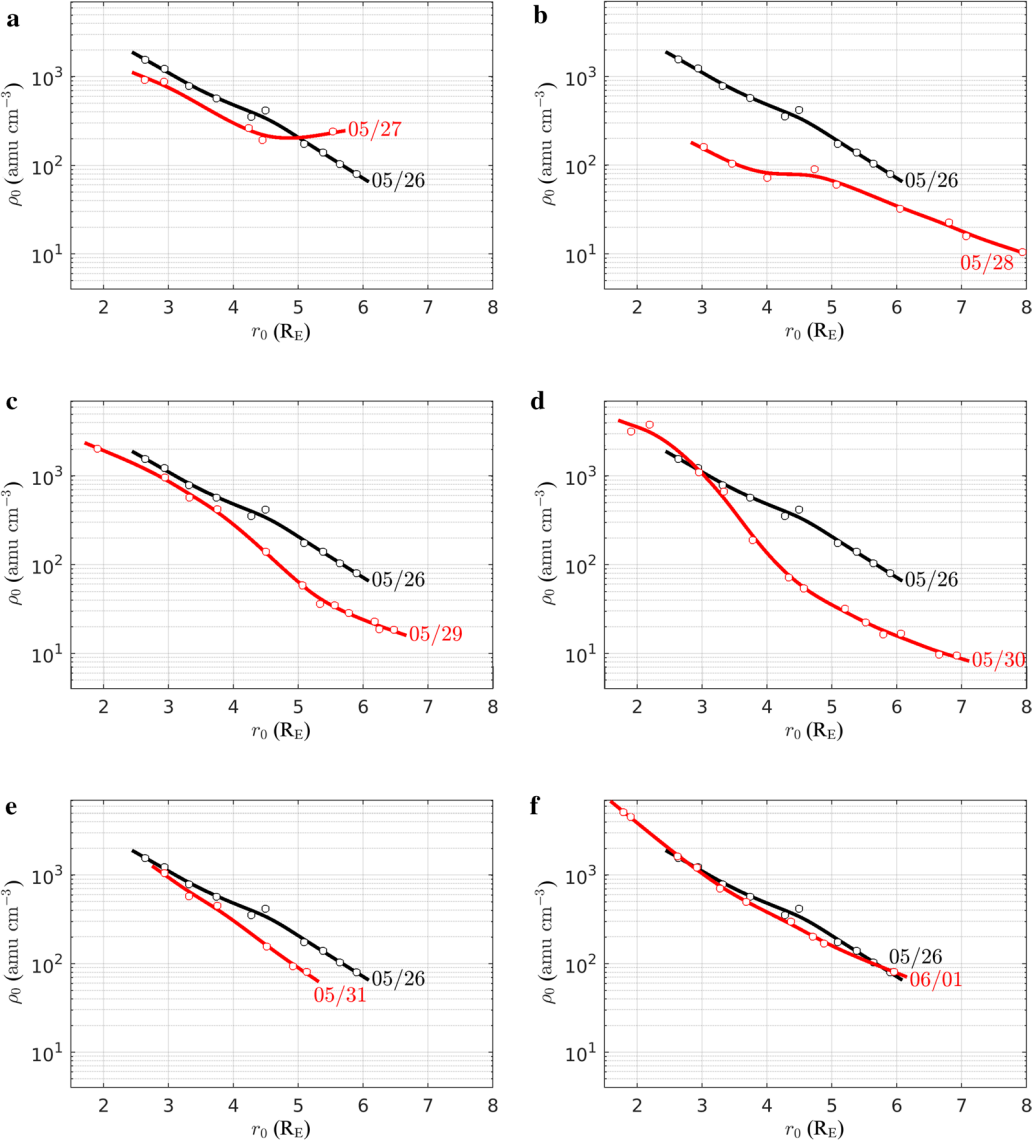
The same as Fig. 8 but at 12:00 UT (~ 14:00 LT)

This latitudinal dependence can be more conveniently described by analyzing the radial profiles of the plasma mass density at different local times as presented in Figs. 8 and 9. These profiles are derived using plasma mass densities inferred from a total of 20 station pairs, namely KEV-IVA, KIL-MUO, MAS-SOD, MUO-PEL, MUO-RAN, PEL-RAN, PEL-OUJ, RAN-OUJ, RAN-HAN, OUJ-HAN, HAN-NUR, NUR-TAR, TAR-BRZ, BRZ-SUW, SUW-BEL, BEL-ZAG, ZAG-VYH, VYH-THY, THY-LOP and RNC-AQU. They represent the equatorial density variation along the direction identified by the average local time of the EMMA stations at a given time (LT ~ UT + 2). The radial variation of the plasma density, shown for LT = 08:30 (Fig. 8) and LT = 14:00 (Fig. 9), is representative of the pre- and post-noon sectors, respectively. Panels from (a) to (f) of each figure show the radial profiles from 27 May to 1 June 2017. The radial profile of 26 May 2017 is superimposed on each panel to highlight deviations from the pre-storm conditions. Open circles are the experimental points, while solid lines are smoothing splines drawn to guide the reader’s eye. The radial profiles show similar behaviors for both LT sectors. The main difference is in the density magnitude that is generally higher in the post-noon sector.

We focus on Fig. 9, which has better data coverage, to describe the plasmasphere dynamics. On 27 May 2017, the profile is approximately similar to that of 26 May 2017 confirming the idea that the plasmasphere is saturated. Nonetheless, some deviations at low and high latitude occur. The results for 28 May 2017 clearly disclose the effects of the storm-enhanced convection. The plasmasphere erosion is visible at all geocentric distances ≥ 3 R_E_ and is particularly pronounced for *r*_0_ < 4 R_E_. This behavior suggests that a plasmapause occurs at geocentric distance lower than 3 R_E_. On 29 May 2017, the plasmasphere apparently recovers to pre-storm conditions up to ~ 3.5 R_E_, while the erosion persists for *r*_0_ > 5 R_E_. It is worth noting that in the morning sector of the same day (panel c of Fig. 8), the recovery seems to be even more pronounced at 4.3 R_E_ (see the red circle just below the black curve), although the proximity of the plasmapause to the region mapped by that station pair might produce a larger error in the estimated density (Milling et al. 2001; Menk et al. 2004). Anyhow, the almost full recovery observed on 29 May 2017 at 3–4 R_E_ seems to be too quick to be due to a refilling from the ionosphere (Rasmussen et al. 1993; Reinisch et al. 2004). It is instead more plausible that during 29 May 2017 EMMA was partially aligned with a corotating plasmaspheric drainage plume which developed during the storm (Goldstein and Sandel 2005). This idea is confirmed by the plasmapause test particle (PTP) simulation (Goldstein et al. 2014), which is available online at http://enarc.space.swri.edu/PTP/.

On 30 May 2017 (panel d of Figs. 8, 9), a new density decrease is observed for *r*_0_ > ~3.5 R_E_, possibly due to a re-enhancement of the magnetospheric convection occurring between 29 May and 30 May 2017, in agreement with the *k*_p_ increase (Fig. 7).

Lastly, the 31 May and 1 June 2017 profiles show a progressive recovery of the plasmasphere at all geocentric distances.

## Ionospheric response

### Ionospheric current system during the sudden impulse

**Fig. 10:**
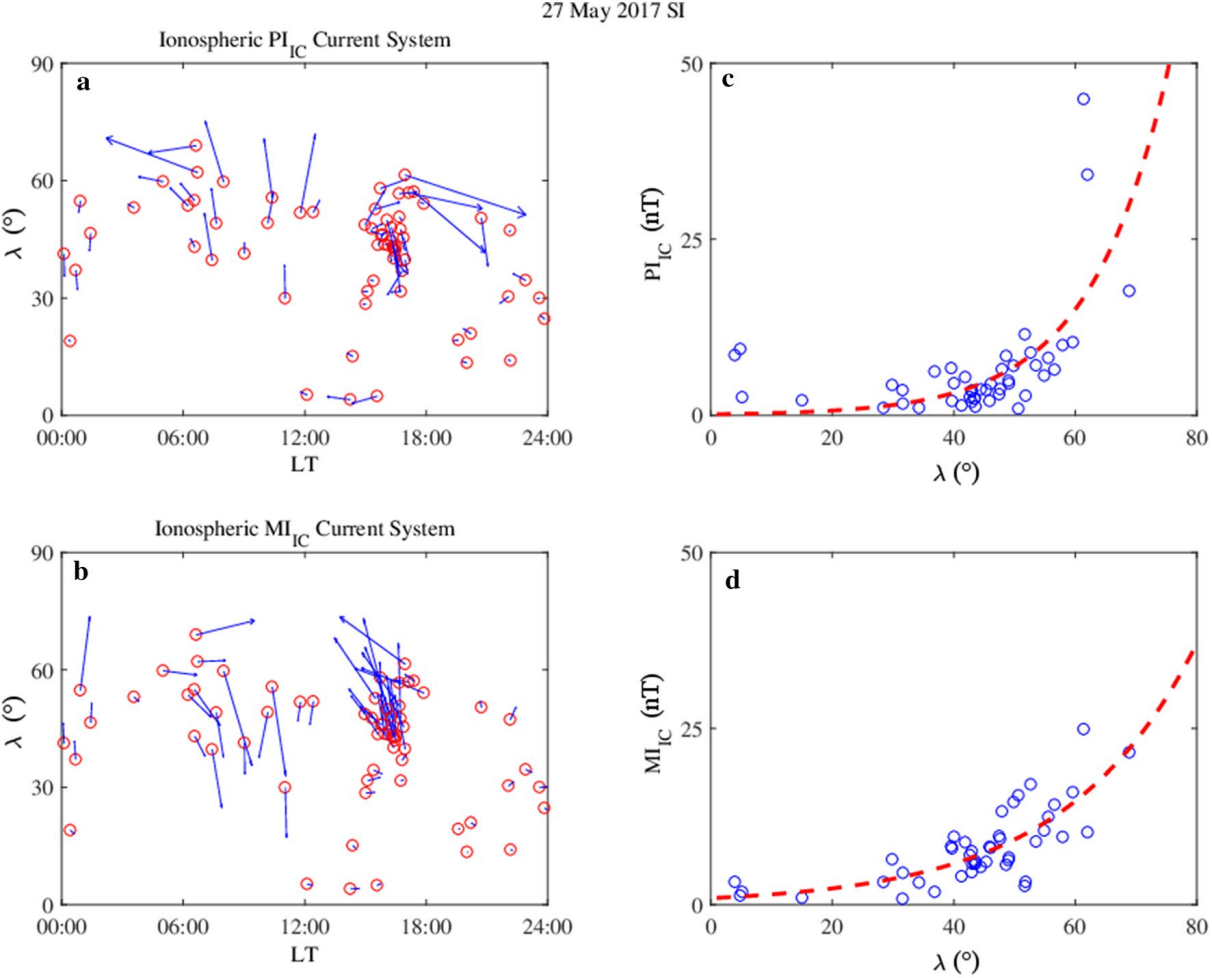
**a**, **b** The ionospheric current directions (blue arrows) as obtained on 27 May 2017 for PI_IC_ at 15:33 UT and for MI_IC_ at 15:42 UT, versus geomagnetic latitude (*λ*) and LT, after a 90° rotation of the DP fields. Red circles correspond to the geomagnetic location of the INTERMAGNET stations. Corresponding amplitudes (blue circles) in the dayside sector (06 < LT < 18) are shown in **c**, **d** versus *λ*, with corresponding exponential fits as red dashed curves. The local enhancement observed around the magnetic equator, which is particularly evident in the PI_IC_ amplitude, is probably due to the equatorial electrojet effect (Carter et al. 2015, 2016)

Figure 10 shows the ionospheric current directions as obtained on 27 May 2017, for PI_IC_ (panel a) at 15:33 UT and for MI_IC_ (panel b) at 15:42 UT, versus the geomagnetic latitude (*λ*) and LT. The behavior of PI_IC_ is consistent with a morning counterclockwise vortex and an afternoon clockwise vortex, while that of MI_IC_ with a morning clockwise vortex and an afternoon counterclockwise vortex. The locations of the vortex foci are at (*λ* ~ 56°, LT ~ 05:40) and at (*λ* ~ 58°, LT ~ 16:40). At low latitudes, PI_IC_ currents are mostly oriented toward west, while MI_IC_ currents toward east, in accordance with Araki (1994) and with Piersanti and Villante (2016). In addition, in the dayside sector, both PI_IC_ and MI_IC_ field amplitudes (panels c and d in Fig. 10) increase exponentially with *λ*; red dashed lines represent the exponential function with the following characteristics: PI_IC_(*λ*) = PI_0_·e^*A*·*λ*^, MI_IC_(*λ*) = MI_0_·e^*B*·*λ*^, with PI_0_ = 0.14 nT, *A* = 0.077 deg^−1^ and MI_0_ = 0.91 nT, *B* = 0.046 deg^−1^. Here, PI_0_ and MI_0_ are the PI_IC_ and MI_IC_ amplitudes derived at *λ* = 0°.

### Ionospheric response in terms of vertical, top and bottom TEC

**Fig. 11:**
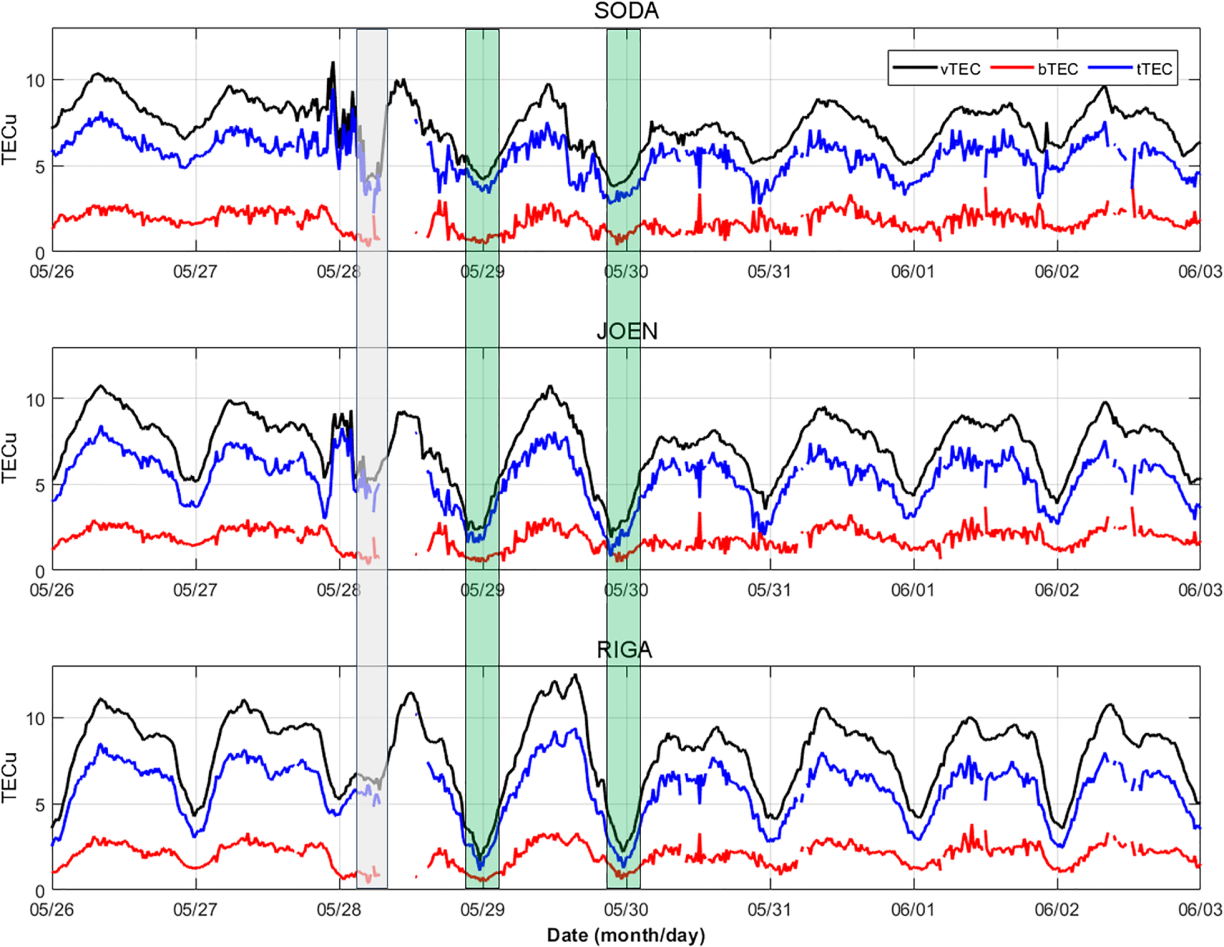
*vTEC* (in black) as measured at SODA (67.4°N, 26.4°E), JOEN (62.4°N, 30.1°E) and RIGA (56.9°N, 24.1°E), from 26 May to 2 June 2017, in UT. SODA, JOEN and RIGA are the closest GNSS receivers to the pairs of magnetometers MUO–RAN, RAN–HAN and TAR–BRZ of the EMMA array, respectively, used to describe the latitudinal dependence of the plasmasphere dynamics as shown in Fig. 7. Corresponding values of *bTEC* (in red), as calculated through the IRI UP method, and *tTEC* = *vTEC* − *bTEC* (in blue), are also displayed. The gray shaded rectangle highlights the significant depletion of *vTEC* occurring in the first half of 28 May 2017, while the green ones highlight the very low values of *vTEC*, recorded during the nights between 28 and 29 May 2017 and between 29 and 30 May 2017, supporting the ionospheric trough movement to lower latitudes, then to lower *L*

Figure 11 shows the patterns obtained for *bTEC*, *tTEC* and *vTEC* at SODA, JOEN and RIGA, from 26 May to 2 June 2017. *tTEC* and *bTEC* are characterized by a lack of data on 28 May 2017, from about 06:00 UT to about 15:00 UT; this is due to the fact that corresponding ionograms are characterized by the G condition, namely a condition for which the critical frequency of the F2 layer, *fo*F2, is equal or lower than the critical frequency of the F1 layer, *fo*F1 (Lobzin and Pavlov 2002; Deminov et al. 2011).

Figure 11 shows that, after the SI, the most important feature is the significant depletion of *vTEC* occurring in the first half of 28 May 2017 (gray shaded rectangle in Fig. 11). Negative phases like these at F-layer heights are thought to be caused by neutral composition changes (Buonsanto 1999; Fuller-Rowell et al. 2013; Olwendo et al. 2017; Piersanti et al. 2017a), specifically, by a decrease in the [O]/[N_2_] ratio, which results in a strong enhancement of the ion loss rate (Prölss 1995). In fact, the SW dissipation energy affects the density structure of the polar upper atmosphere by increasing the heavier gases (especially the molecular nitrogen N_2_) and decreasing the lighter gases (especially the atomic oxygen O). Anomalous increases in the [N_2_]/[O] density ratio are a permanent feature of the polar thermosphere, but during more active conditions, these composition perturbations intensify and expand toward lower latitudes. This initial negative phase is then followed by a sudden increase in *vTEC* values, which is attributable to the “dusk effect,” a characteristic of the dusk sector at latitudes where the plasmasphere–ionosphere coupling is more pronounced (Prölss 1995; Buonsanto 1999; Mendillo 2006). Interpretations of this effect are summarized by Prölss (1995) and Buonsanto (1999), and, as they highlighted, competing theories related to neutral winds (sudden onset of strong equatorward winds from auroral heating) and electrodynamics (prompt appearance of electric fields of magnetospheric origin) can each give the necessary plasma uplift to regions of reduced loss, even though the magnetospheric convection seems to play the most important role. The positive phase related to the dusk effect is terminated by the F-layer trough (Moffett and Quegan 1983; Rodger et al. 1992; Krankowski et al. 2009) movement to lower *L* values after sunset, caused by the plasmasphere contraction. These dynamical reasons of *vTEC* negative phases are rather different than those related to an enhanced loss via chemistry, which are dominant during daytime. The fact that the ionospheric trough moves to lower latitudes and then to lower *L* is supported by the very low values of *vTEC* recorded during the nights between 28 and 29 May 2017 and between 29 and 30 May 2017 (green shaded rectangles in Fig. 11). Since our plasmasphere observations are limited to daytime hours, it is not possible to directly compare the plasmapause location with the nighttime ionospheric trough; nonetheless, the plasmasphere observations in the post-noon sector on 28 May 2017 (panel b of Fig. 9) indicate indeed an earthward motion of the plasmapause to *L* < 3, which maps to a latitude lower than that of RIGA station.

**Fig. 12:**
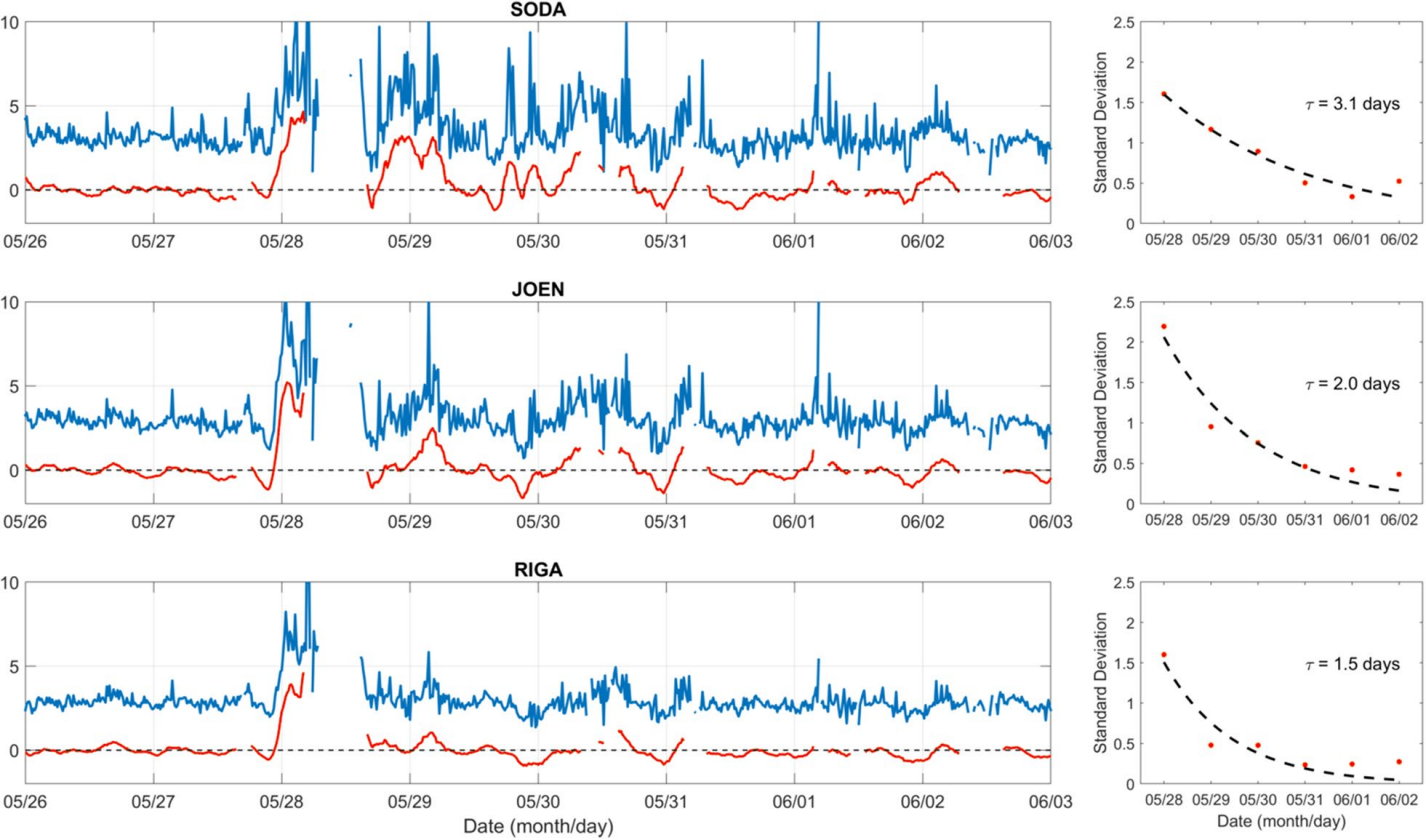
The ratio *tTEC*/*bTEC* (in blue) as calculated at SODA (67.4°N, 26.4°E), JOEN (62.4°N, 30.1°E), and RIGA (56.9°N, 24.1°E), from 26 May to 2 June 2017, in UT. SODA, JOEN and RIGA are the closest GNSS receivers to the pairs of magnetometers MUO–RAN, RAN–HAN and TAR–BRZ of the EMMA array, respectively, used to describe the latitudinal dependence of the plasmasphere dynamics as shown in Fig. 7. Corresponding 2 h and half running means to which the mean value of 26 May 2017, considered as the quiet time reference, has been subtracted, are also displayed in red. In the right panels, the daily standard deviations related to the red curves, from 28 May to 2 June 2017, are plotted and fitted through an exponential function *y* = *A*·e^(−*t*/*τ*)^, where *τ* is a characteristic time typifying the recovery phase of the ionospheric plasma

The daytime *vTEC* values recorded on 29 May 2017 at SODA and JOEN are comparable with those recorded on 26 May 2017, which is considered here as the quiet reference day; on the contrary, *vTEC* values recorded on 29 May 2017 at RIGA are instead higher than those recorded on 26 May 2017. This is likely due to an enhanced fountain effect, which gives rise to a poleward expansion of the northern crest of the equatorial ionization anomaly (Liu et al. 2008; Balan et al. 2010; Zong et al. 2010; Cesaroni et al. 2017; Piersanti et al. 2017a), which prevails the plasma decrease caused by neutral composition changes. This means that instead at SODA and JOEN, where daytime *vTEC* values are comparable to those recorded on 26 May 2017, the two mechanisms counterbalance. In the next 4 days, between the 30 May and the 2 June 2017, SODA, JOEN and RIGA are characterized each by a daytime *vTEC* negative phase, which means that during these days the daytime plasma decrease caused by the neutral composition change is prevailing. Figure 11 shows that *tTEC* and *bTEC* trends are similar to that of *vTEC*, while the corresponding ratio *tTEC*/*bTEC* shown in Fig. 12 is somewhat different, the higher the latitude, the greater the ratio. This is understandable on the basis that the F-layer neutral composition changes are more effective at higher latitudes, where the dissipation of solar wind energy occurs. This means that going from lower to higher latitudes the negative phase characterizing the F-layer, and hence the bottomside part of the vertical electron density profile, is more pronounced and consequently the ratio *tTEC*/*bTEC* is higher. This is even clearer looking at the red curve of Fig. 12, which is a two hours and half running mean of the *tTEC*/*bTEC* time series to which the mean value of 26 May 2017, considered as the quiet time reference, has been subtracted.

In order to evaluate a characteristic time of the recovery phase of the ionospheric plasma, the daily standard deviations related to these red curves, from 28 May to 2 June 2017, are calculated and fitted through an exponential function *y* = *A*·e^(−*t*/*τ*)^, where *τ* is the ionospheric characteristic time we are looking for (see right panels of Fig. 12). Going from higher to lower latitudes, the value of *τ* decreases from 3.1 to 1.5 days, giving a simple quantitative evidence that, during the recovery phase, the redistribution of the ionospheric plasma between the bottomside and the topside part of the vertical electron density profile is slower at higher latitudes.

## Discussion and conclusions

In this work, it has been investigated how the ICME occurred on 27 May 2017 affected the ionosphere–magnetosphere–plasmasphere system. Even though the main declared intention of the paper is to investigate the plasmaspheric depletion and see whether and how is related to the ionospheric dynamics, to have a comprehensive view of the phenomenon, the interplanetary conditions, the magnetosphere response in terms of the magnetopause motion, and the ionospheric current flow pattern have been also analyzed.

According to the estimated shock characteristics, the IS front of the event under investigation is expected to impact the magnetopause at 15:38 UT (~ 57 min after WIND observations) first in the pre-noon region. The SI onset starts instead five minutes earlier (at ~ 15:33 UT). This feature, as well as the slow rise in the magnetic field components corresponding to the SI as recorded by GOES satellites, could depend on both the inclination of the IS front (Wang et al. 2006) and a value of *V*_Sh_ higher than the one estimated (~ 349 km/s).

In the post-noon region, THEMIS E observed the SI almost simultaneously to GOES, while THEMIS D, located closer than THEMIS E to the magnetopause boundary, detected a clear jump 3 min later than the other satellites. This behavior can be explained considering a propagation of the disturbance from the pre-noon region to the post-noon region according to the estimated impact point. In fact, the jump in the *B*_*z*_ component of the magnetospheric field is greater at GOES13 (the nearest to the shock impact point), decreases at similar values at both GOES15 (dawn region) and THEMIS E (post-noon region) and assumes the lowest value at THEMIS D (dusk region). These magnetospheric field variations are quite well represented by the TS05 model for each spacecraft in terms of the coupling between the magnetopause current and the ring current. Typically, the expected result for a magnetospheric sudden impulse, in terms of a magnetospheric currents ignition, is the turning on of the magnetopause current alone (Araki 1994; Villante and Piersanti 2008; Piersanti and Villante 2016). In this case, the combined effect of the strong magnetospheric compression, driven by the SW dynamic pressure enhancement (and testified by the signature of multiple magnetopause crossings recorded by THEMIS A and THEMIS E), and of the southward turning of the IMF, which consequently increased the ion energy density of the ring current (Roeder et al. 1999; Kane 1974), gives rise to the activation of both the magnetopause and the ring currents.

The principal effect of the magnetospheric SI is the generation of an ionospheric DP-2 cell current, which completely modifies the dynamics and the geometry of the ionospheric current flows observed during a solar quiet period (Chapman 1929; Matsushita and Maeda 1965; De Michelis et al. 2010; Alberti et al. 2016; Piersanti and Villante 2016, 2017a). Our results show a DP-2 cell current characterized by: a PI_IC_ current vortex flowing counterclockwise in the morning and clockwise in the afternoon; a MI_IC_ current vortex flowing clockwise in the morning and counterclockwise in the afternoon. These results are in agreement with Araki (1994) and Piersanti and Villante (2016).

The GS related to the ICME was preceded by a period of very quiet geomagnetic activity that lasted for at least 3 days. This prolonged quiet condition allowed the plasmasphere to reach a state of quasi-saturation. The effects of the main phase of the storm are clearly visible on 28 May 2017, when the observed plasma density generally falls by a factor of ~ 5–10. A definite estimation of the magnitude of the erosion was not possible due to the lack of data in the morning sector and in general at low latitudes, although an upper limit of 3 R_E_ for the plasmapause position can be deduced from the post-noon radial density profile of 28 May 2017. An analysis of observations from Swarm satellites (Friis-Christensen et al. 2006), using the technique by Heilig and Lühr (2018), also indicates a midnight location of the plasmapause at *L* ~ 2.5 at the beginning of 28 May 2017 (not shown).

During the recovery phase, the plasmasphere shows the typical latitudinal dependent features resulting by the concurring processes of magnetospheric convection and refilling from the ionosphere. At low geocentric distances (up to ~ 3.5 R_E_), the plasmasphere reached the pre-storm conditions in ~ 1 day, while at higher distances the recovery stood for at least ~ 4 days. However, the fast recovery at *L* < 3.5 is probably not due to a refilling from the ionosphere. Both theoretical predictions (e.g., Rasmussen et al. 1993) and previous experimental observations (Park 1974; Obana et al. 2010) evidence indeed that the process of flux tube refilling from the ionosphere takes at least 3 days at *L* = 3. As pointed out by Denton et al. (2016), the observed density variation from day to day may not refer to the same flux tube because the action of the convection electric field between two successive measurements may cause strong departures from a pure plasma corotation. For example, the convection of a plasmaspheric drainage plume into the viewing area of EMMA during 29 May 2017 might well account for the observed density increase.

A second minor enhancement of the geomagnetic activity, and therefore of the magnetospheric convection, occurred on the night between 29 and 30 May 2017, causing further erosion for *r*_0_ > 4.5 R_E_.

The ionospheric response shows the typical latitudinal behavior characterized by greater variations at higher latitudes. The *tTEC*/*bTEC* ratio shows a significant increase during the very beginning of the main phase of the GS, mainly due to a decrease in *bTEC* caused by neutral composition changes. During the recovery phase, this ratio comes back to the pre-storm conditions with a characteristic time showing a direct dependence on latitude. At *L* ~ 3, the characteristic time is ~ 1.5 days and increases to ~ 2 days at *L* ~ 4 and to ~ 3.1 days at *L* ~ 5. This feature is comparable to the one observed for the plasmasphere recovery which, for similar *L* values, varies from ~ 1 to more than 4 days. It is, however, not possible to identify a direct relation between the plasmaspheric refilling and the ionospheric top/bottom recovery to pre-storm conditions. In fact, while the ionospheric recovery time is mainly driven by the reestablishment of the bottom side neutral composition to pre-storm conditions, the plasmaspheric recovery time, as pointed out above, is strongly affected by the convection electric field variation, especially for flux tubes mapping at high latitudes. Nonetheless, a reduction of *vTEC*, as observed between 30 May and 2 June 2017 at each latitude, could significantly influence a slower plasmaspheric refilling rate due to a reduction of the supplied plasma (Villante et al. 2006). This is, however, a feature that deserves to be further and more deeply investigated by considering a dataset of GSs of different intensity for which there is the same wealth of available data.

## Data Availability

Ionosonde data used in this study are publicly available at the Digital Ionogram Database (http://ulcar.uml.edu/DIDBase/) and can be freely downloaded by means of the SAO Explorer software developed by the University of Massachusetts, Lowell (http://ulcar.uml.edu/SAO-X/SAO-X.html). RINEX files were downloaded from the EUREF Permanent GNSS Network at http://www.epncb.oma.be/. Geomagnetic indices were downloaded from the OMNIWeb Data Explorer—NASA site at https://omniweb.gsfc.nasa.gov/form/dx1.html. WIND and THEMIS data were downloaded at https://cdaweb.sci.gsfc.nasa.gov/index.html/. GOES data were downloaded at https://www.swpc.noaa.gov/. The TS05 model and IRI model codes are, respectively, available at http://geo.phys.spbu.ru/~tsyganenko/modeling.html and http://irimodel.org/. Swarm data were downloaded through the ESA Web site https://earth.esa.int/web/guest/swarm/data-access. EMMA data are not publicly available; anyhow the authors are available to share them under request. INTERMAGNET data were downloaded at www.intermagnet.org. The datasets generated and/or analyzed during the current study are available from the corresponding author on reasonable request.

## Abbreviations

EMMA: European quasi-Meridional Magnetometer Array
FACs: field-aligned currents
FLR: field line resonance
GS: geomagnetic storm
GSE: geocentric solar ecliptic system
GSM: geocentric solar magnetic system
ICME: interplanetary coronal mass ejection
IMF: interplanetary magnetic field
IRI: International Reference Ionosphere
IRI UP: International Reference Ionosphere Update
IS: interplanetary shock
LT: local time
PTP: plasmapause test particle
SI: sudden impulse
SW: solar wind
ULF: ultra-low frequency
UT: universal time
